# A behavioral and electrophysiological investigation of conflict monitoring in cystinosis (CTNS gene mutations) using the flanker paradigm

**DOI:** 10.3389/fneph.2026.1608421

**Published:** 2026-03-12

**Authors:** Sophie Molholm, Ana A. Francisco, Douwe J. Horsthuis, Tringa Lecaj, Dennis Cregin, Chloe Brittenham, John J. Foxe

**Affiliations:** 1 The Cognitive Neurophysiology Laboratory, Department of Pediatrics, Albert Einstein College of Medicine, Bronx, NY, United States; 2 Department of Neuroscience, Rose F. Kennedy Center, Albert Einstein College of Medicine, Bronx, NY, United States; 3 The Frederick J. and Marion A. Schindler Cognitive Neurophysiology Laboratory, Ernest J. Del Monte Institute for Neuroscience & Department of Neuroscience, University of Rochester School of Medicine and Dentistry, Rochester, NY, United States

**Keywords:** executive function, EEG, lysosomal storage disorder, N2, P3

## Abstract

Cystinosis, a rare lysosomal storage disease, is characterized by cystine crystallization and accumulation within tissues and organs, including the kidneys and brain. Its impact on neural function appears mild relative to its effects on other organs, but therapeutic advances have led to substantially increased life expectancy, necessitating deeper understanding of its impact on neurocognitive function. Behaviorally, some deficits in executive function have been noted in this population, but the underlying neural processes are not understood. Using standardized cognitive assessments and a Flanker task in conjunction with high-density electrophysiological recordings (EEG), we investigated the neural dynamics of conflict monitoring in individuals with cystinosis, when compared to age-matched controls. Thirty-six individuals diagnosed with cystinosis (8–38 years old, 25 women) and 39 age-matched controls (23 women) participated in this study. As expected, slower reaction times and larger amplitudes were observed in incongruent vs congruent trials in both groups, suggesting largely maintained conflict monitoring in cystinosis. However, when compared to their age-matched peers, individuals with cystinosis presented larger differences between congruent and incongruent trials both behaviorally (reaction times) and electrophysiologically (N2, P3). Our findings suggest that individuals with cystinosis are able to monitor and adapt to conflict, even if slower, less accurately, and more effortfully than their age-matched peers.

## Introduction

Cystinosis, a rare autosomal recessive disorder, results from mutations in the CTNS gene on chromosome 17p13.2 (1–3). Through excessive accumulation and crystallization of cystine within lysosomes, CTNS mutations cause widespread damage to various tissues and organs, including the brain (4–8). Structural brain differences (9–13) and difficulties related to cognitive function (14–18) have been described in association with CTNS mutations.

Despite having shown larger visual evoked potentials (19) and some differences in sensory memory and attentional processes (20, 21), our prior electrophysiological (EEG) work in cystinosis revealed generally intact sensory processing and no extensive neural differences (20, 21). Though encouraging, this set of findings belies the poor performance on standardized neurocognitive assessments and the difficulties with school work that are commonly seen in individuals with cystinosis (22). Our previous work was, however, primarily focused on early sensory auditory and visual processing and did not explore downstream cognitive complex processes, more likely to explain such differences and difficulties. One set of skills of interest that could indeed relate to poor performance in academic and professional contexts falls under executive functioning.

Executive function describes the set of control processes that govern goal-directed behavior and serve to optimize performance on complex cognitive tasks, allowing one to behave flexibly and to adapt to novel, changing circumstances (23). Executive functions, which relate to a broad range of responses—from basic and immediate responses to slower ones, influenced by long-term planning (24)—undergo gradual and continuous development that extends into adulthood (24, 25). While only three behavioral studies have explored executive function in cystinosis, findings indicate some difficulties in this population (15, 16, 26), though not consistently. Using the Delis-Kaplan Executive Function System (D-KEFS) Trail Making Test, Verbal Fluency Test, Design Fluency Test, Color-Word Interference Test, and Sorting Test Ballantyne et al. demonstrated poorer performance across these executive functioning components among children and adolescents with cystinosis (15); Besouw et al. observed differences only in sustained attention, not in other executive functioning components (16).

Here, we focused on conflict monitoring, a process that detects conflict and initiates control processes to resolve said conflict. Conflict monitoring involves two key elements: a monitoring component that assesses the level of conflict and a control adaptation component that adjusts attentional filters based on current task demands. While the former appears to be associated with the dorsal regions of the anterior cingulate cortex, the latter is likely linked to activity in the dorsolateral prefrontal cortex (27–30). These components work in tandem—the conflict signal prompts control adaptation, indicating the need for attentional changes; subsequent adjustments in attention result in a reduction of conflict (29). Behaviorally, children living with cystinosis seem to present difficulties monitoring conflict (15). But the neural mechanisms of conflict monitoring have not yet been investigated in this population and thus it is unclear at what stage of the conflict monitoring process such difficulties arise.

Conflict monitoring is commonly measured with tasks in which ignoring distracting cues is crucial for adequate performance. In the current study, we employed a behavioral color-word interference task (from the Delis-Kaplan Executive Function System, D-KEFS; 31) and an EEG Flanker task (32). In the color-word interference task, one expects that the presence of conflict results in longer completion times and more errors. In the classic Flanker task, participants indicate the direction of a centrally presented target arrow that is flanked by arrows that are either directionally congruent (pointing in the same direction) or directionally incongruent (pointing in the opposite direction). The flanker congruency effect, i.e., slower and less accurate responses in incongruent versus congruent trials, is expected behaviorally and represents conflict related processing. Electrophysiologically, the presentation of a stimulus inducing conflict typically results in a more prominent negative event-related potential (ERP) occurring fronto-centrally 200–300 ms after stimulus presentation (N2) and a positive ERP roughly 150 ms later with a centro-parietal topography (P3) (33, 34). The response inhibition evoked N2, attributed to neural activity from anterior/mid cingulate cortex and inferior frontal cortices, is associated with conflict detection and resolution (35). Its amplitude correlates with task difficulty, being more negative for more challenging tasks (33). The P3 reflects stimulus evaluation and decision-making processes (36) and indexes cognitive control and attentional allocation in conflict resolution (37). P3 sources include a complex interplay of cortical regions specifically tailored to the demands of cognitive conflict resolution and attentional allocation of a given task and primarily involve the anterior cingulate, frontal, and parietal lobes (e.g., (38, 39)).

The effective resolution of conflicting information, a crucial aspect of adaptive behavior and decision-making, requires extra executive function resources. We argue that this additional taxing of the system could be particularly impactful to those with cystinosis. Individuals with cystinosis may thus present, behaviorally, longer completion and reaction times and more errors and, electrophysiologically, a different modulation of the ERP components N2 and P3 when compared to their age-matched peers. A better characterization of the cognitive profile associated with cystinosis is critical to developing effective interventions to compensate for or improve areas of cognitive vulnerability.

## Materials and methods

### Participants

We enrolled 36 individuals diagnosed with cystinosis (age range: 8–38 years old, 25 women) and 39 age-matched controls (23 women). Individuals with cystinosis were recruited via social media and through contact with family organizations. Due to the rareness of cystinosis, most participants, all of whom lived within the United States, traveled from out-of-state to participate. Controls were recruited from the local community using flyers near our research facility, e-screens in the medical school and hospital, and our existing participant database. While we verbally screened for neurological and learning differences, we did not explicitly screen the control group for conflict-monitoring difficulties.

Exclusion criteria for controls included a history of developmental or educational difficulties or delays, neurological issues, or a severe mental illness diagnosis. Individuals with cystinosis were excluded if they had current neurological problems or a severe mental illness diagnosis. Visual acuity was assessed using a Snellen chart, and all participants had either normal or corrected-to-normal vision. Nevertheless, at the beginning of the EEG session, all participants were asked if they could see the stimuli and their components without difficulty. Consent was obtained from all participants, with legal guardians signing for those under 18. Participants were compensated monetarily for their time. This study and its associated procedures received approval from the Albert Einstein College of Medicine Institutional Review Board and adhered to the principles of the Declaration of Helsinki.

### Experimental procedure and stimuli

Participation consisted of three visits—one visit dedicated to cognitive function assessments, the other two to EEG recordings.

The cognitive function battery employed included the assessment of, among other abilities, verbal and non-verbal intelligence (using age-appropriate Wechsler Intelligence Scales (40, 41)), working memory (assessed with the Stanford-Binet Intelligence Scales, SB-5 (42)), and executive function components (reported here, the Color-Word Interference test of the Delis-Kaplan Executive Function System, D-KEFS (31)). Standardized scores were the dependent measures for the Wechsler Intelligence Scales and the Stanford Binet. For the Color-Word Interference test, the measure used here is calculated by contrasting performance on the interference condition (C3) with performance on the color naming condition (C1), providing a standardized metric of conflict effects. The inhibition condition (C3) assesses the ability to inhibit the prepotent response of reading the word when the color of the ink differs from the color named by the word itself. Potential color naming differences are then controlled for by using the C3 vs. C1 contrast. Scaled scores normed by age are reported. Scaled scores have an average score of 10 and a standard deviation of 3, with higher scores representing better performance.

A traditional (EEG) flanker task (32) was developed to examine the neural mechanisms associated with conflict monitoring. Participants were presented with stimuli consisting of a row of five simple arrows on the computer screen. The arrows pointed either to the left or to the right. Participants were instructed to respond to the central target arrow. If the arrow pointed to the left, they were asked to press a key on the left side of the keyboard with their left index finger; alternatively, if the arrow pointed to the right, they were asked to press a key on the right side of the keyboard with their right index finger. Different hands were used to avoid confusion for our younger participants. Since we did not have hypotheses about which hand was used to respond, or whether the arrow was pointing rightward or leftward, for subsequent analyses the data were collapsed across right and left hand (and arrow) responses. The stimulus lasted 200 ms, and the interstimulus interval (ISI) varied randomly between 1500 and 2000 ms. On congruent trials, all flanking arrows pointed in the same direction as the center arrow (either < < < < <, or > > > > >), whereas on incongruent trials, the central target arrow pointed in one direction and the flanking arrows point in the opposite direction (either < < > < <, or > > < > >). The different types of congruent and incongruent flanker stimuli were shown with equal probability. To ensure task comprehension subjects practiced on 20 trials and were given feedback. Accuracy and reaction times were the behavioral dependent measures.

### Data acquisition and analysis

Continuous EEG data were acquired from 64 scalp electrodes at a sampling rate of 512 Hz (Active 2 system; Biosemi™, The Netherlands; 10–20 montage). Data preprocessing was performed using the EEGLAB toolbox (version 2023.0) (43) for MATLAB (version 2023a; MathWorks, Natick, MA). The complete processing pipeline is available at: https://github.com/DouweHorsthuis) (44). The preprocessing steps involved down-sampling the data to 256 Hz, re-referencing it to the average reference, and applying a 1 Hz high-pass filter with a 0.5 Hz transition bandwidth and a filter order of 1690, as well as a 45 Hz low-pass filter with an 11 Hz transition bandwidth and a filter order of 152. Both filters were implemented as zero-phase Hamming windowed sinc FIR filters. Noisy channels were identified and removed using the Clean Rawdata plugin in EEGLAB, followed by visual confirmation. Artifacts stemming from blinks and saccades were mitigated through Independent Component Analysis (ICA). Additionally, the spherical spline method was employed to interpolate channels that had been removed during the earlier steps. Data were segmented into epochs of -50 ms to 600 ms and a baseline of -50 ms to 0 ms applied. An automatic artifact rejection criterion (moving window peak-to-peak threshold at 120 μV) was applied. In the control group, 2.12% of trials were, on average, excluded. In the cystinosis group, the average exclusion rate was 4.25%. One participant with cystinosis had more than 30% of trials rejected and was therefore excluded from the analyses. The number of trials included in the analyses differed between groups (CT: M = 452.85, SD = 36.54, CYS: M = 423.88, SD = 67.43, *p* = .03, *d’* = 0.54). For the final analysis only trials with correct responses were included.

There was significant variability in performance across participants, and performance was poorer for incongruent trials. Balancing the cost-benefit ratio between maximizing the number of participants included and maintaining a reasonable signal-to-noise ratio, to equate the number of trials per group, we carried out a random selection of 100 congruent trials and 50 incongruent trials per participant and ran the statistical models on those subsets.

#### Behavioral data

The proportion of correct responses and average reaction time in congruent and incongruent trials were calculated per subject. A mixed-effect model was used for the reaction times; an independent sample t-test was used to test for differences in accuracy between the groups. Lastly, we tested for correlations between neural components, behavioral responses, and standardized cognitive measures. P-values derived from t-tests and correlations underwent Holm-Bonferroni corrections to account for multiple comparisons (45). This correction was executed using the p.adjust function from the stats package in R (46).

#### Electrophysiological data

Frontal and central channels (Fz, FCz, and Cz) were included for N2 and P3 analyses. Windows of interest were between 280 and 380 ms for the N2, and 400 and 500 ms for the P3. These windows and scalp regions corresponded to N2/P3 in prior flanker studies and captured the modulations in the grand mean data (47). Mixed-effects models were constructed using the lmer function in the lme4 package (48) in R (46). Within these models, group and trial (congruent vs incongruent) were designated as fixed factors, while participants were included as random factors. Model fitting was carried out based on the maximum likelihood criterion, and p-values were calculated using Satterthwaite approximations.

Fixed-effect coefficients (β) from LMMs are reported as effect sizes, reflecting differences in ERP amplitude (µV) associated with experimental factors while accounting for trial-level variance and random effects.

## Results

### Demographics and cognitive function measures

**Table 1** shows a summary of the included participants’ age and biological sex. Two-sample independent-means *t* tests were run in R (46) to test for group differences. In cases in which the assumption of the homogeneity of variances was violated, *Welch* corrections were applied to adjust the degrees of freedom. There were no differences between control and cystinosis groups in age, sex, verbal IQ, or in the Color-Word interference test (CWIT) contrast 1 scaled score. The full set of CWIT scaled scores are presented in an appendix for the interested reader. Note that CWIT was missing for 7 control and 1 cystinosis participant. The groups differed in nonverbal IQ and nonverbal working memory, with individuals with cystinosis presenting lower scores than their age-matched peers. Handedness was reported for most participants (all but three: one control adult, one cystinosis adult, and one cystinosis child). Of these, all were right-handed except for one cystinosis adult, one cystinosis child and one control child.

**Table 1:** Demographic and cognitive characterization of the individuals included in the analyses.

	Control	Cystinosis	Statistical test	Effect size
Age	M=19.28; SD = 8.56	M=20.00; SD = 8.84	*t* = -0.35, *df* = 70.57, *p* = .76	*d* = 0.08
Sex	23 F, 16 M	25 F, 10 M	χ*^2^* = 0.77, *df* = 1 *p* = .76	w=0.13
Verbal IQ	M=101.94; SD = 12.77	M=96.94; SD = 10.43	*t* = 1.72, *df* = 58.23, *p* = .36	*d* = 0.43
Nonverbal IQ	M=103.84; SD = 11.02	M=89.43; SD = 11.55	*t* = 5.20, *df* = 63.56, *p* = .01	*d* = 1.28
Nonverbal WM	M=10.19; SD = 2.47	M=8.03; SD = 3.60	*t* = 2.88, *df* = 59.05, *p* = .03	*d* = 0.70
CWIT-Interference contrast 1 (	M=11; SD = 2.44	M=11.65; SD = 2.35	*t* = -1.11, *df* = 64.70, *p* = .27	*d* = 0.27

### Flanker task

#### Behavioral performance

**Figure 1:**
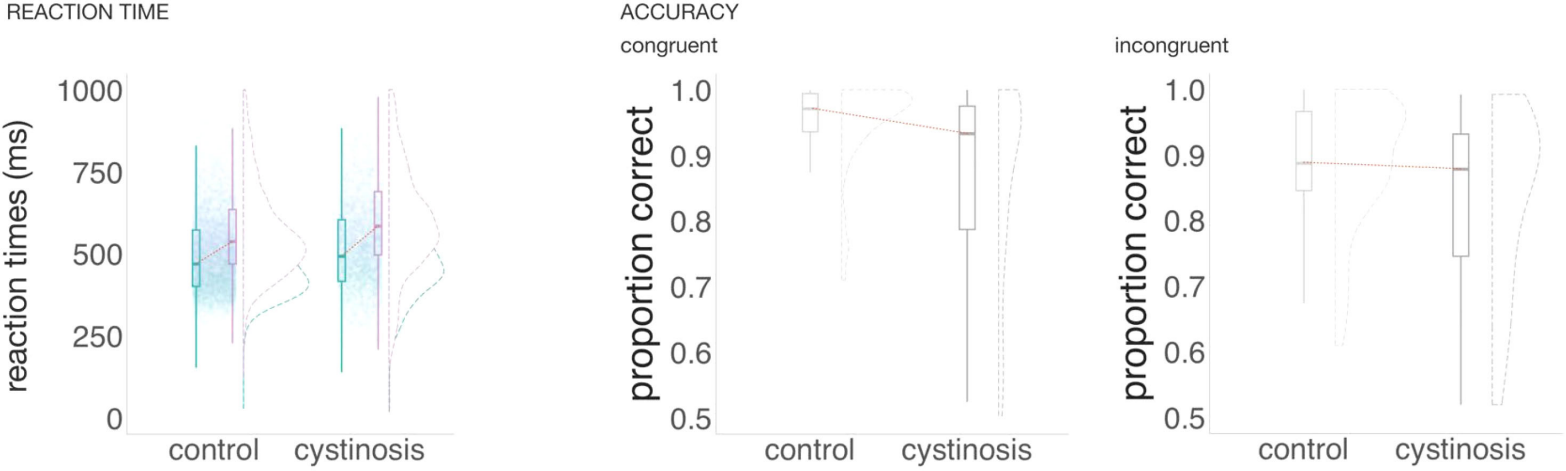
Average reaction time and proportion of accuracy per group and type of trial.

**Figure 1** shows the participants’ behavioral performance (reaction times and accuracy) for congruent and incongruent trials. To test for differences between the groups in reaction time, mixed-effects models were implemented as described above. The interaction between group and type of trial was significant (*ß* = 10.78, SE = 4.17, *p* = .01): The difference in reaction time between incongruent and congruent trials was larger in the cystinosis group when compared to the control group. Response times were slower in incongruent than in congruent trials (*ß* = 81.50, SE = 2.70, *p* = .01). The significant group by trial type interaction reflects a moderate group difference in behavioral congruency effects, with cystinosis showing an approximately 11-ms larger incongruent–congruent reaction time cost relative to controls, alongside a robust overall slowing for incongruent trials. To test for differences in accuracy between the groups, two-sample independent-means *t* tests were run in R (46). When compared to their age-matched peers, individuals with cystinosis presented a lower proportion of correct responses in both congruent (*t* = 3.24, *df* = 41.78, *p* = .01, *d* = 0.79) and incongruent trials (*t* = 3.22, *df* = 50.28, *p* = .01, *d* = 0.77). The difference in accuracy between congruent and incongruent trials was not different between the groups (*t* = 0.84, *df* = 49.38, *p* = .41, *d* = 0.20).

#### ERPs

**Figure 2:**
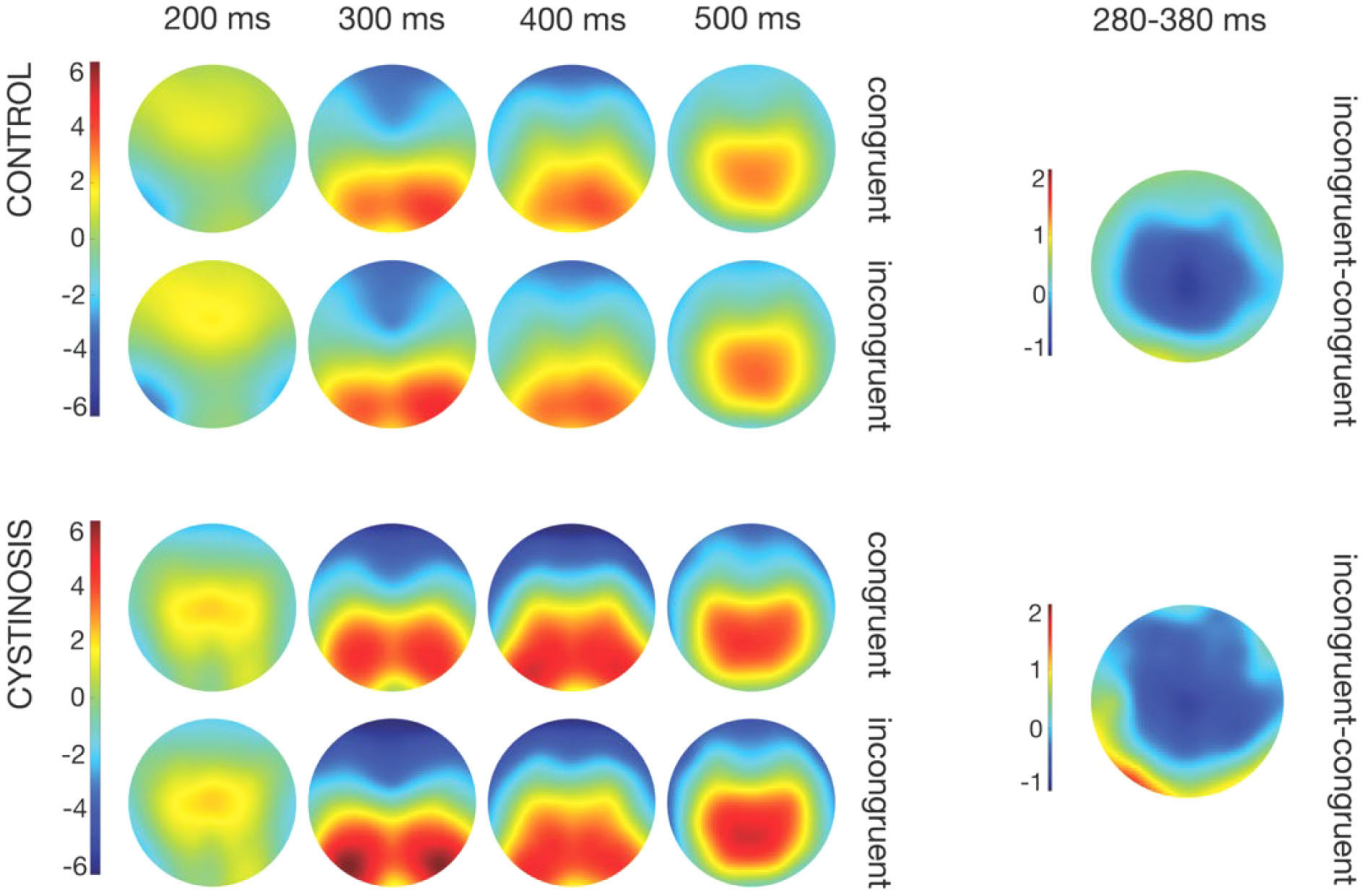
Topographic maps per group and type of trial and topographic difference between incongruent and congruent trials per group.

**Figure 3:**
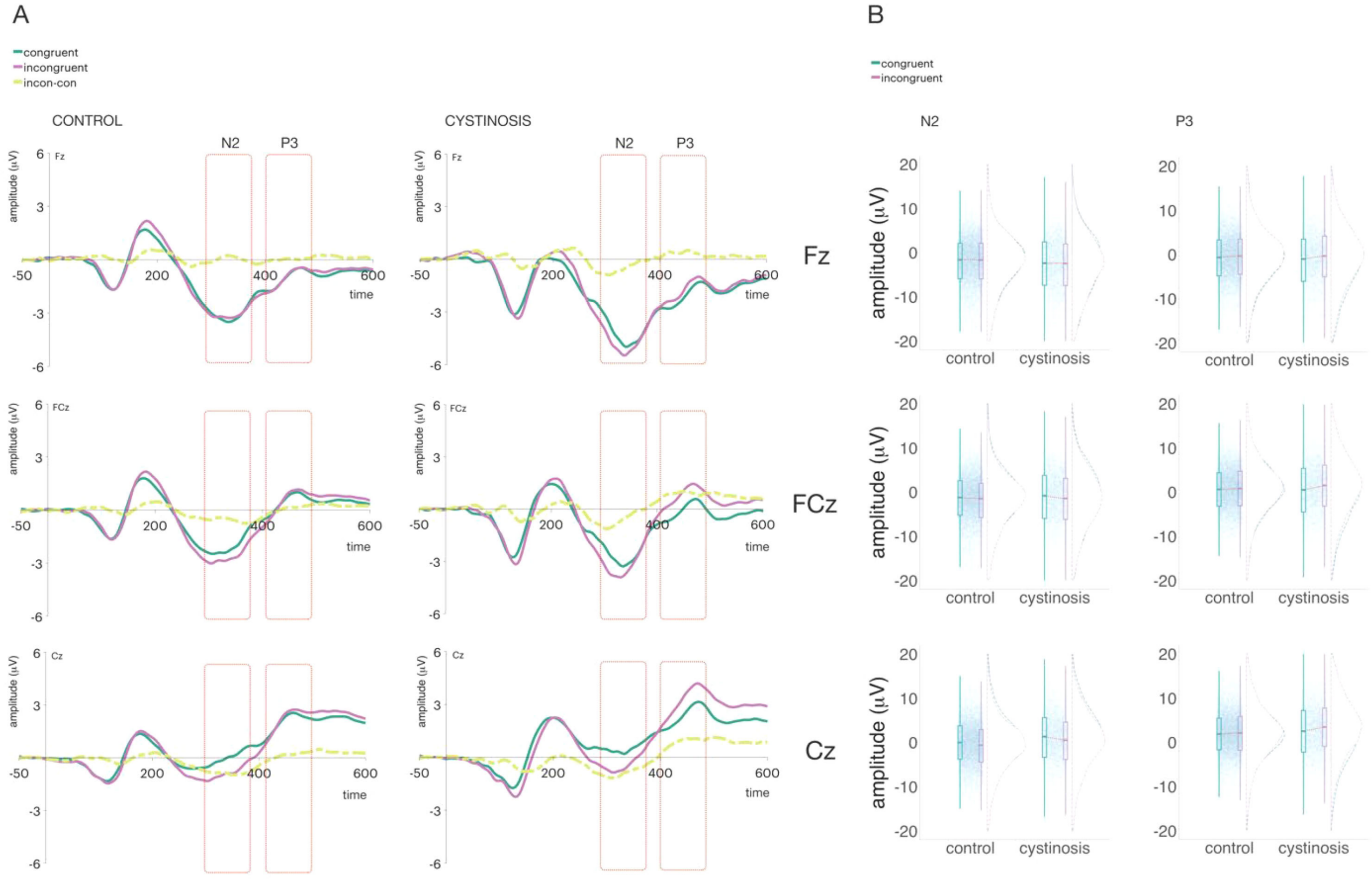
**(A)** Averaged ERPs per group and trial type at Fz, FCz, and Cz. **(B)** Plots showing distribution of amplitudes (trial-by-trial data) for the time windows of interest.

**Figure 2** shows topographic maps between 200 and 500 ms per group and trial type for activity resulting from the difference between incongruent and congruent trials for control and cystinosis groups. The groups appear to show similar topographies. Amplitudes seem to be, however, slightly larger in the cystinosis group, which can be likewise observed in **Figure 3**, which focus on the ERPs of interest (**Figure 3**: N2 and P3).

In the N2 time window (see **Figures 3A, B**), the interaction between group and trial type was significant in FCz: The difference between congruent and incongruent trials was larger in those with cystinosis than in the control group (*ß* = 1.14, SE = 0.37, *p* = .01). This interaction reflects a 1.14 µV greater differentiation between incongruent and congruent trials in cystinosis relative to controls. In both FCz and Cz, incongruent trials evoked more negative components than congruent trials (FCz: *ß* = -1.11, SE = 0.24, *p* = .01; Cz: *ß* = -0.77, SE = 0.22, *p* = .01), corresponding to moderate condition differences in N2 amplitude across groups.

In the P3 time window (see **Figures 3A, B**), the interaction between group and trial type was significant in FCz and Cz. As for the N2, the difference between congruent and incongruent trials was larger in those with cystinosis than in the control group (FCz: *ß* = 1.49, SE = 0.36, *p* = .01; Cz: *ß* = 0.95, SE = 0.35, *p* = .01), indicating a substantially amplified trial-type modulation in cystinosis during later processing stages.

## Discussion

Individuals living with cystinosis present difficulties in tasks that rely on executive functioning abilities (22). Utilizing standardized cognitive measures and EEG recordings, we asked whether individuals with cystinosis show, when compared to controls, differences in conflict monitoring—an important component of executive function and a crucial aspect of adaptive behavior and decision-making. We expected that, behaviorally, those with cystinosis would take longer to respond and complete tasks and make more errors. Additionally, we hypothesized that N2 and the P3 would be differently modulated in cystinosis.

The expected flanker congruency effect was observed in individuals with cystinosis and controls, that is, both groups presented slower and less accurate responses to incongruent versus congruent trials and larger N2 and P3 amplitudes in incongruent compared to congruent trials. One could thus argue that conflict monitoring appears to be largely maintained in cystinosis. This is consistent with intact color-word interference task performance compared to controls for this group of cystinosis participants. And, indeed, both behaviorally and electrophysiologically, the group differences are moderate.

Behaviorally, both groups were slower on incongruent trials compared to congruent trials. The difference in response time between incongruent and congruent trials was, however, larger in the cystinosis group than in the control group. **Figure 1** suggests that although individuals with cystinosis were overall slower regardless of trial type (consistent with evidence of slower processing speed in this population (16, 49, 50), the incongruent trials distribution in the cystinosis group appears more skewed to longer reaction times. This enlarged difference is consistent with more effortful processing in cystinosis when monitoring conflict. Notably, incongruent trials imposed only a moderate additional reaction time burden (≈11 ms) beyond that observed in controls. Different from what was observed for reaction times, the difference in accuracy between congruent and incongruent trials was similar between the groups. Thus it appears that in dealing with increased cognitive demands under conflict conditions, accuracy was prioritized over reaction times in the cystinosis group. However, individuals with cystinosis showed large reductions in accuracy overall, regardless of type of trial (d ≈ 0.8), indicating a broad performance disadvantage that extends beyond conflict-specific demands. Similarly, in previous studies utilizing different paradigms, we reported overall lower accuracy rates in cystinosis (e.g., (49)). It is worth considering whether the overall differences in accuracy that we have observed in cystinosis relate to methodological choices. To guarantee that a specific response corresponds to one stimulus and not to the one that follows it, responses are only considered in a specific time window following stimulus presentation. As individuals with cystinosis show slower response times, it is possible that they are not responding less accurately, but that their answers are more often outside of the time window that is being considered. We think this is unlikely, however. As illustrated in **Figure 1**, the proportion of responses at the maximal end of the reaction-time window is very small, regardless of group, suggesting that most responses fell within the response window used for data analysis.

Similar to what was observed for reaction times, when compared to their age-matched peers, individuals with cystinosis showed larger differences between incongruent and congruent trials in the N2 and P3 brain responses. The pattern of brain responses in N2/P3 time windows appear, however, similar between individuals with cystinosis and their age-matched peers, suggesting, once again, that conflict monitoring is relatively maintained in cystinosis—even if individuals need more time and make more errors when detecting conflict. As for the enlarged difference between congruent and incongruent trials, consistent with the reaction time findings, conflict monitoring may be more effortful in cystinosis, and thus more cognitive resources need to be allocated. It is notable that for this stimulus configuration, in which congruent and incongruent trials were equally probable, P3 only modulated for the cystinosis group. The moderate-to-large group differences observed in our data indicate meaningfully amplified neural differentiation between incongruent and congruent trials in cystinosis relative to controls.

While N2 has been associated with conflict detection (35), the classic P3 has been argued to reflect stimulus evaluation and decision-making processes (36, 51) and index cognitive control and attentional allocation in conflict resolution (37). In previous studies, we argued for differences in attention (19, 20) and memory (21) in cystinosis. Consistent with this idea, the flanker effect is often attributed to insufficient attentional filtering of flankers, which leads to competing response activations in the case of incongruent trials (e.g., (52)). Attentional difficulties in cystinosis could thus call for additional resources when monitoring conflict, leading to increased flanker conflict effects. The contribution of potential attention and memory difficulties in cystinosis, which may have contributed to the lower nonverbal IQ and nonverbal working memory scores in the present data, should be considered when drawing conclusions about conflict monitoring. Executive functioning is not unitary. Rather, it comprises separable components (53, 54) such as task-switching, updating, and information monitoring. Each of these components may be recruited across a variety of tasks including selective attention, decision-making, working memory, error monitoring, conflict monitoring, and language processing (53, 55, 56). It is thus difficult to disentangle how such components interact and to design experimental paradigms that tap into the different processes in exclusive ways. The brain regions involved in solving executive functioning type tasks are, likewise, multiple and their associations complex. Here we only manipulate one type of executive function, conflict monitoring. Additional research is clearly needed to 1) understand the landscape of executive functioning in cystinosis, and 2) how this changes over development. Network analysis and graph theory applied to a broad set of executive functioning tasks could shed some light on how different components inform each other in cystinosis.

In summary, while some differences were found between individuals with cystinosis and a group of controls while responding to a traditional conflict monitoring task, our findings suggest that individuals with cystinosis are able to monitor conflict, even if slower, more effortfully, and less accurately than their age-matched peers. That individuals with cystinosis were generally slower and made more mistakes during this conflict monitoring is, nevertheless, relevant when defining strengths and devising best practices to support academic and professional development in this population. Of note, non-verbal and visual-spatial difficulties are often much more prevalent in this population than verbal differences, as our current findings confirm. As slower and/or less accurate monitoring of conflict could impact some of the academic difficulties observed in cystinosis, training the ability to monitor and manage response conflict (and filter out irrelevant information) or other related processes such as attentional filtering may be impactful and generalize to other related executive functioning components.

This study is not without its limitations. First, the groups were not matched for IQ. While we elected not to include IQ as a covariate in our statistical analyses given its close association with the diagnostic status, this decision may nonetheless have influenced the observed outcomes, as differences in cognitive ability could contribute to variability in performance across groups. Second, variables related to current health status (such as a measure of renal function) and compliance to treatment, which has been linked to better clinical outcomes (57), were not included in the present study but could be useful in understanding group- and individual-level differences in a larger sample study. Relatedly, however, most individuals who participated in the study were relatively healthy due to the requirement to travel relatively long distances (for most participants) to participate and thus, our sample is not representative of the full spectrum of individuals living with cystinosis. It is possible that individuals with greater cystinosis-related symptoms experience greater challenges in executive functions such as conflict monitoring. Third, whereas our data span a large age range that includes both children and adults, our participant numbers are not sufficient to power examination of the developmental trajectory of the inhibitory processes of interest. While developmental changes would be expected based on well-characterized shifts in connectivity across the neural networks engaged by executive functions (58), assessing if this developmental trajectory is altered in cystinosis is challenging due to the rarity of the condition. Nevertheless, it is important that future work will target this question. It is also important to consider the effect sizes and the sensitivity of the paradigm employed. While we chose to balance the proportion of congruent and incongruent trials per block, others have shown the visual flanker effect to be larger in blocks in which the proportion of congruent trials is high compared to low (see (59) for a review). Still, behavioral and electrophysiological conflict effects were present in both groups and differed between groups. Future work systematically manipulating congruent/incongruent stimulus probabilities could help to understand how increasing levels of conflict are managed by individuals with cystinosis. Lastly, although real-life situations often require engagement in more than one task at a time (60), here we focused on a single-item task. As we gather more evidence on the cognitive functioning profile characteristic of cystinosis, it is crucial to shed more light on whether specific systems have limits after which effectiveness is unmanageable or whether it is the accumulation of slight differences in separate components and their interactions that result in the cognitive difficulties described in cystinosis.

## Data Availability

The raw data supporting the conclusions of this article will be made available by the authors, without undue reservation.
