# Supplementary material for: A behavioral and electrophysiological investigation of conflict monitoring in cystinosis (CTNS gene mutations) using the flanker paradigm
Source: Front Nephrol. 2026 Mar 12;6:1608421. doi: 10.3389/fneph.2026.1608421 (PMC13017271; doi:10.3389/fneph.2026.1608421)
Supplement: Supplementary file 1 [file Table1.docx]

| Condition/contrasts | **control** | **cystinosis** | **statistical test** | **effect size** |
| --- | --- | --- | --- | --- |
| **Condition 1 (Scaled)** | M=9.48; SD=3.24 | M=7.56; SD=3.14 | *t*=-2.47, *df*=64.74, *p*=.02 | *d*=0.60 |
| **Condition 2 (Scaled)** | M=10.79; SD=2.16 | M=8.65, SD=2.96 | *t*=-3.39, *df*=60.39, *p*<.01 | *d*=0.82 |
| **Condition 3 (Scaled)** | M=10.48; SD=2.71 | M=9.21; SD=3.45 | *t*=1.69, *df*=62.26, *p*=.10 | *d*=0.41 |
| **Condition 4 (Scaled)** | M=10.24; SD=2.81 | M=8.15; SD=2.35 | *t*=2.80, *df*=64.53, *p*=.01 | *d*=0.68 |
| **Inhibition vs. Color Naming** | M=11.00; SD=2.44 | M=11.65; SD=2.35 | *t*=-1.11, *df*=64.70, *p*=.27 | *d*=0.27 |
| **Inhibition/Switching vs. Combined Color Naming+Reading** | M=9.88; SD=2.68 | M=9.85; SD=3.01 | *t*=0.04, *df*=64.53, *p*=.97 | *d*=0.01 |
| **Inhibition/Switching vs. Inhibition** | M=9.45; SD=2.68 | M=8.94; SD=3.03 | *t*=0.73, *df*=64.45, *p*=.47 | *d*=0.18 |

Appendix 1. Condition and contrast scores from the Color-Word Interference test of the Delis-Kaplan Executive Function System
